# Кетогенная диета: история возникновения, механизм действия, показания.

**DOI:** 10.14341/probl12724

**Published:** 2021-11-03

**Authors:** Е. В. Иванникова, М. В. Алташина, Е. А. Трошина

**Affiliations:** Российский геронтологический научно-клинический центр; Национальный медицинский исследовательский центр эндокринологии; Национальный медицинский исследовательский центр эндокринологии

**Keywords:** низкоуглеводная диета, кетодиета, углеводы, сердечно-сосудистые заболевания

## Abstract

Кетогенная диета уже более 100 лет весьма успешно применяется с целью компенсации течения ряда тяжелых неврологических заболеваний. В последние годы стремительно растущее число пациентов с ожирением и сахарным диабетом 2 типа заставляет искать новые эффективные способы снижения массы тела. Применение кетогенной или низкоуглеводной диеты в данном ключе вызывает повышенный интерес как среди врачей, так и населения в целом. Более того, публикации результатов новых исследований позволяют предположить, что подобный рацион может играть терапевтическую роль не только при ожирении, но и при других патологиях, например, синдроме поликистозных яичников или злокачественных новообразованиях. Тем не менее данная модель питания с резким ограничением доли углеводов на фоне повышенного содержания жира не является сбалансированной, а долгосрочная безопасность ее применения, как и влияние в первую очередь на риски развития сердечно-сосудистых заболеваний, до сих пор остаются малоизученными. В данной статье представлены описание различных вариантов кетогенной диеты, механизм ее действия, а также результаты наблюдения пациентов на фоне низкоуглеводного рациона как в краткосрочном периоде, так и с оценкой отдаленных последствий.

## Abstract (en)

Low-carb diets have been successfully used to alleviate a number of severe neurological diseases for about 100 years. The publication of the results of new studies suggesting that this type of diet may play a therapeutic role in other pathologies such as diabetes, obesity, polycystic ovary syndrome, and oncology is of particular interest for both doctors and the public. However, the long-term safety of using a low-carb or ketogenic diet, as well as its impact, primarily on the risks of developing cardiovascular diseases, remains poorly studied. This article presents the results of observation of patients against the background of a low-carbohydrate diet, both in the short-term perspective as well as provides an assessment of its long-term consequences.

## ВВЕДЕНИЕ

Хорошо известно, что для снижения массы тела достаточно сократить потребление энергии и увеличить ее расход. При внешней легкости этот шаг представляет собой трудно решаемую задачу: за последние 40 лет число людей с ожирением возросло в 2 раза [1]. Доказано, что для эффективного снижения веса необходим междисциплинарный подход: лечение должно включать индивидуальный подбор питания, применение психотерапевтических техник изменения пищевого поведения, различные терапевтические и хирургические методы [2]. Несмотря на существующие лечебные программы, только 20% людей способны достичь цели и поддерживать 10% снижение веса в течение года, большинство же возвращаются к исходным данным в среднем за 3–5 лет [2]. Одним из спорных вопросов является выбор рациона, поскольку до сих пор нет однозначного мнения о том, какой диетический протокол самый эффективный и безопасный как в краткосрочной, так и долгосрочной перспективе [3]. Ранее наиболее часто рекомендации по питанию пациентам с ожирением включали в себя относительно высокое содержание трудноусвояемых углеводов на фоне сниженной доли жира. Однако, согласно некоторым исследованиям, ограничительные режимы питания приводят лишь к умеренному и непродолжительному успеху, поскольку для большинства пациентов придерживаться подобных моделей питания длительное время проблематично. В связи с чем в последние годы поиск «универсальной» диеты приобрел направленность в сторону снижения потребления углеводов.

Согласно рекомендациям Всемирной организации здравоохранения (ВОЗ) по содержанию нутриентов, питание взрослого человека должно состоять на 55–75% из углеводов, 10–15% белков и 15–30% жиров [4]. Считается, что такие пропорции удовлетворяют потребность в макроэлементах человека среднего возраста с умеренным уровнем физической активности без тяжелых сопутствующих заболеваний. Кетогенная диета (КД) представляет собой лечебный рацион с потреблением углеводов ниже рекомендованных 50–75% (обычно ниже 150 г углеводов в день или 13% общего потребления энергии, по данным разных источников) и относительным увеличением доли жира (может достигать 70–80%) и белка (как правило, 10–20%) [5]. Примерный расчет для рациона на 2000 калорий представляет собой 165 г жира, 40 г углеводов и 75 г белка. Тем не менее КД не следует рассматривать как диету с высоким содержанием белка, поскольку его ежедневное потребление составляет примерно 1,2–1,5 г/кг идеальной массы тела, что не превышает норму потребления белка взрослого человека.

Следует отметить, что «стандартной» схемы КД с определенным соотношением между белками, жирами и углеводами в настоящее время не существует. В литературе отмечается некоторая путаница с определением КД, поскольку многие исследователи предлагают разделять на кетогенные низкоуглеводные диеты, включающие 50–150 г углеводов, и кетогенные, которые содержат 50 г углеводов максимум [6].

Подобное деление обусловлено прежде всего разной эффективностью применения вариантов КД при лечении ожирения. Так, в ходе проведения сравнительного метаанализа значительной разницы по динамике снижения веса на фоне низкоуглеводного (где углеводы составили до 45% суточной калорийности) и стандартного рационов получено не было, в отличие от данных пациентов с более значимым (до 50 г/сут) ограничением углеводов [5]. Однако необходимо учитывать, что проводить подобные исследования весьма сложно прежде всего из-за отсутствия возможности контроля соблюдения участниками рекомендаций по питанию. Еще сложнее интерпретировать полученные результаты, поскольку проведение количественной оценки потери жировой ткани может проводиться различными методами с неодинаковой чувствительностью.

Золотым стандартом оценки доли жировой и тощей массы тела является двухэнергетическая рентгеновская абсорбциометрия (DXA) в режиме Total Body [7]. Однако, часто применяются и другие методы: антропометрический и метод непрямой калориметрии, а также инструментальная диагностика: биоимпедансометрия, компьютерная и магнитно-резонансная томография. При этом некоторые авторы считают, что даже таким высокоточным методам, как DXA, может не хватать чувствительности для фиксирования минимальных изменений объема жировой ткани [8]. Этот момент может играть важную роль при проведении сравнительной оценки эффективности диет с разной углеводной нагрузкой. Особое внимание стоит уделить и срокам наблюдения пациентов, поскольку только недавно стали появляться данные долгосрочного наблюдения приверженцев разных вариантов питания [9].

## ИСТОРИЯ ВОЗНИКНОВЕНИЯ НИЗКОУГЛЕВОДНОЙ ДИЕТЫ

Первые данные о терапевтическом эффекте КД были опубликованы в 1797 г. при описании ее успешного применения у солдат, больных сахарным диабетом (СД) и проявлениями глюкозурии [10]. Рацион состоял из «молока, известковой воды (limewater), хлеба с маслом, кровяной колбасы, мяса и топленого жира». Интересны упоминания родственника хорошо известного всем эндокринологам Фредерика Бантинга (Frederick Banting), Уильяма Бантинга (William Banting) [11]. В своем «письме о тучности, адресованном общественности» от 1864 г. имеющий ожирение, однако успешно похудевший Уильям Бантинг (William Banting) описывал свою диету с ограничением потребления «хлеба, масла, сахара, пива и картофеля». Письмо стало настолько популярно, что даже появился термин «banting», означающий отказ от чего-то вкусного. В шведском языке «banta» по-прежнему является основным глаголом, который переводится как «сидеть на голодной диете».

В 1921 г. Роллин Тернер Вудьят (R. T. Woodyatt) сообщил, что на фоне голодания и высокожировой диеты наблюдалось накопление кетоновых тел (КТ). На основе этих данных Рассел Морс Уайлдер (Russell Morse Wilder) впервые ввел термин «кетогенная диета» для высокожирового рациона (60–75% жира, 15–30% белка, 10% углеводов) как одного из возможных методов лечения эпилепсии за счет увеличения циркулирующих КТ, обладающих противосудорожным эффектом [12]. С древних времен было известно, что длительное голодание или «водная диета» могут приводить к снижению частоты припадков при эпилепсии  [13]. Возможно, самым первым упоминанием о подобном действии низкоуглеводного рациона (по сути голодания) является описанное в Новом Завете «чудесное» исцеление (Новый Завет, Матфея 17: 14–21).

Когда они пришли к народу, то подошел к Нему человек и, преклоняя пред Ним колени,

сказал: Господи! помилуй сына моего; он в новолуния беснуется и тяжко страдает, ибо часто бросается в огонь и часто в воду,

я приводил его к ученикам Твоим, и они не могли исцелить его.

Иисус же, отвечая, сказал: о, род неверный и развращенный! доколе буду с вами? доколе буду терпеть вас? приведите его ко Мне сюда.

И запретил ему Иисус, и бес вышел из него; и отрок исцелился в тот час.

Тогда ученики, приступив к Иисусу наедине, сказали: почему мы не могли изгнать его?

Иисус же сказал им: по неверию вашему; ибо истинно говорю вам: если вы будете иметь веру с горчичное зерно и скажете горе сей: «перейди отсюда туда», и она перейдет; и ничего не будет невозможного для вас;

сей же род изгоняется только молитвою и постом.

Педиатр Муни Густав Петерман (Mynie Gustav Peterman) предложил применять принцип КД и у детей из расчета 1 г белка на 1 кг массы тела и не более 10–15 г углеводов в день [13]. Диета оказалась весьма успешной: в 1925 г. автор сообщил, что у 95% из 37 маленьких пациентов приступы наблюдались реже, а у 60% вообще прекратились. К 1930 г. был накоплен опыт наблюдения уже около 100 подростков и взрослых. Однако были отмечены не только положительные эффекты КД (улучшение концентрации внимания, поведения и сна), но и побочные (тошнота и рвота из-за избыточного кетоза), в связи с чем появление противоэпилептических препаратов в 1940-х гг. отодвинуло терапию КД на второй план. Лишь в 1990-х гг. КД вновь стала применяться в качестве одного из способов лечения тяжелых форм эпилепсий и других неврологических заболеваний. Снижение частоты приступов на фоне КД, согласно результатам некоторых исследований, может достигать 24% [14].

## ВАРИАНТЫ НИЗКОУГЛЕВОДНОЙ ДИЕТЫ

В настоящее время наряду с оригинальной КД существует несколько модификаций, таких как диеты low-carbohydrate-high-fat и moderate-carbohydrate. Кроме того, Джорджем Л. Блэкберном (George L. Blackburn) была введена концепция «модифицированной диеты с сохранением белка» (protein-sparing modified fast (PSMF)) — строгого ограничительного режима питания (менее 800 ккал в сутки), основанного в первую очередь на потреблении минимального количества белка, необходимого для сохранения безжировой массы тела и нацеленного на достижение быстрой потери веса.

Далее — подробнее о наиболее популярных вариантах КД (табл. 1).

**Table:** Таблица 1. Условная классификация вариантов кетогенных диет

Название	Содержание углеводов*	Содержание белков*	Содержание жиров*
VLCKD или VLCD с очень низким содержанием углеводов	<10% 20–50 г/сут	10% 1,2–1,5 г/кг	70–80%
LCD или LCHF низкоуглеводная, богатая жирами	<10–26% >50, но <130 г/сут	10–30%	25–45%
Moderate-carbohydrate среднее потребление углеводов	26–44% 100–170 г/сут	10–30%	25–35%
Классическая КД	3%	7% *	90%

VLCKD (very low carb ketogenic diet) — КД с очень низким содержанием углеводов

Первоначально классический вариант КД был основан на принципе 4:1, где преобладающим источником энергии являлись жиры (более 90% суточной калорийности на фоне потребления 7% белков и 3% углеводов). Все варианты VLCKD, независимо от того, включают ли они производные животного или растительного происхождения, основаны на строгом ограничении общего потребления углеводов менее 50 г/день. Количество белка ограничено до 1 г/кг массы тела у большинства пациентов и 1,5 г/кг массы тела при активной физической нагрузке [15], то есть практически не отличается от рекомендованных порций. Использование сливок, мороженого и молока не рекомендуется из-за более высокого содержания лактозы. В программах часто предлагается говядина травяного откорма и домашняя птица свободного выгула, которые содержат немного больше ω-3 полиненасыщенных жиров, чем выращенные на зерновом корме, рыба (преимущественно выловленная в холодных водах). Допустимы небольшие порции некоторых фруктов и ягод. Хотя ряд программ и допускает потребление малыми порциями крепких спиртных напитков, низкоуглеводных вин или пива, большинство же рекомендуют их ограничить, как и напитки с добавлением подсластителей.

LCHF (low-carbohydrate-high-fat) — низкоуглеводная КД, богатая жирами

Для LCHF характерно наличие менее 20–26% углеводов от суточной калорийности на фоне потребления более 50% жиров и белков. Под строгим запретом потребление картофеля, риса, хлеба, продуктов на основе муки и кукурузы (в том числе печенья, десертов), сухофруктов, сладких напитков, масел с высоким содержанием ω-6 полиненасыщенных жирных кислот (например, кукурузного, подсолнечного, сафлорового, соевого и арахисового). Допустимы фасоль, чечевица, орехи, фрукты и даже шоколад, но с высоким содержанием какао-бобов (от 65 до 90%)

Moderate-carbohydrate — КД со средним потреблением углеводов

По данным различных источников, в данной диете рекомендуется большее потребление белковой пищи на фоне небольшого содержания в пище насыщенных жиров, свежих овощей и цельнозерновых продуктов. Белки предпочтительно растительного происхождения (10–30% от суточной калорийности).

Также в отдельных источниках упоминается модифицированная диета Аткинса (MAD), диета со средней длиной цепи триглицеридов (MCT) и КД с низким гликемическим индексом (LGIT) [16].

Несмотря на некоторые различия, все низкоуглеводные диеты приводят к аналогичным изменениям гомеостаза.

## МЕХАНИЗМ ДЕЙСТВИЯ НИЗКОУГЛЕВОДНОЙ ДИЕТЫ

Строгое ограничение углеводов в рационе оказывает заметные эффекты на метаболизм и вызывает так называемый «физиологический кетоз» (в отличие от патологического диабетического кетоацидоза) [17]. После нескольких дней голодания или диеты с резким ограничением углеводов (менее 20 г в день) запасы глюкозы в организме становятся недостаточными для производства оксалоацетата и окисления жиров в цикле Кребса. Поскольку единственным «топливом» для центральной нервной системы (ЦНС) человека является глюкоза (жирные кислоты в этом качестве выступать не могут, так как не проникают через гематоэнцефалический барьер), в данной ситуации необходим альтернативный источник энергии [18]. Из периферических жировых отложений мобилизуются свободные жирные кислоты (СЖК). Попадая в печень, СЖК подвергаются митохондриальному ß-окислению с образованием ацетоацетил-КоА и, в конечном итоге, КТ, объединяющих ацетоацетат, ацетон и ß-гидроксибутират (β-HB) [19][20]. Традиционно β-HB рассматривается как главный фактор терапевтического эффекта от терапии КД, который оказывает плейотропное действие и считается индикатором кетоза. Важно подчеркнуть, что печень производит КТ, но не может их использовать из-за отсутствия фермента 3-кетоацил-КоА-трансферазы, необходимого для преобразования ацетоацетата в ацетоацетил-КоА. После секреции в кровоток β-HB становится основным источником энергии для клеток головного мозга и других тканей, где он снова превращается в ацетил-Кo-A и вступает в цикл Кребса [21]. β-HB также играет роль в синтезе холестерина и липогенезе de novo и, согласно новым данным, в процессах, влияющих на транскрипцию генов и окислительный стресс.

Присутствие избытка КТ в крови и их выведение с мочой вызывает кетонемию и кетонурию. В нормальных условиях концентрация КТ очень низка (<0,3 ммоль/л) по сравнению с глюкозой (примерно 4 ммоль/л), и, поскольку глюкоза и КТ имеют аналогичный Km (или константу Михаэлиса–Ментен) для транспорта глюкозы в мозг, КТ начинают использоваться в качестве источника энергии в ЦНС и других тканях, когда они достигают концентрации около 4 ммоль/л, что близко к Km для транспортера монокарбоксилата [22]. При этом гликемия остается в пределах физиологической нормы. Фактически глюкоза образуется из двух источников: в первые дни КД основным источником глюкозы является неоглюкогенез из аминокислот, впоследствии на первый план выходит глицерин, высвобождаемый в результате лизиса триглицеридов (ТГ) [23].

Во время физиологического кетоза кетонемия достигает максимального уровня 7–8 ммоль/л без изменения pH, тогда как при диабетическом кетоацидозе она может превышать 20 ммоль/л с сопутствующим снижением pH крови. Уровни КТ в крови у здоровых людей не превышают 8 ммоль/л именно потому, что ЦНС и другие ткани используют кетоны для получения энергии вместо глюкозы.

## ПОКАЗАНИЯ ДЛЯ НИЗКОУГЛЕВОДНОЙ ДИЕТЫ

Изначально КД была предложена в качестве немедикаментозной сопутствующей терапии ряда неврологических заболеваний, впоследствии список показаний расширился. Согласно рекомендациям Ассоциации Диетологов Италии (Associazione Italiana di Dietetica e Nutrizione Clinica) [5], назначение КД возможно в следующих случаях:

Ряд авторов относят к показаниям для КД также наличие синдрома поликистозных яичников (СПКЯ), врожденного гиперинсулинизма, болезни Паркинсона (БП), болезни Альцгеймера (БА), бокового амиотрофического склероза, мигрени, нарколепсии, депрессии, аутизма, травмы и ишемии, рака/злокачественных новообразований [23].

Однако, несмотря на растущую популярность, мнение исследователей о целесообразности и безопасности применения КД неоднозначно. Например, Европейская ассоциация по изучению ожирения (The European Association for the Study of Obesity, EASO) подчеркивает, что КД может быть использована только как часть комплексной программы с обязательным регулярным наблюдением специалиста в области питания и диетологии [5].

Согласно руководству Национального института здравоохранения и качества ухода Великобритании (National Institute for Health and Care Excellence, NICE), КД следует применять не более 12 нед (непрерывно или периодически) также с обязательным постоянным лабораторным (общий и биохимический анализы крови, тиреотропный гормон, витамин D 25(ОН), общий анализ мочи (с определением кетонурии и микроальбуминурии)) и клиническим контролем [24].

## ПРОТИВОПОКАЗАНИЯ ДЛЯ НИЗКОУГЛЕВОДНОЙ ДИЕТЫ

КД — модель питания, вызывающая кетоз. что может привести к развитию определенных осложнений и ограничений в ее применении, в том числе и при следующих заболеваниях:

Среди побочных эффектов КД выделяют кратковременные, появляющиеся в первые дни после изменения рациона, и долговременные, развитие которых происходит через несколько месяцев. В следующем разделе приведены наиболее значимые последствия КД на организм.

## ВЛИЯНИЕ НА РАЗЛИЧНЫЕ ОРГАНЫ И СИСТЕМЫ

Нервная система

Эпизодические и пароксизмальные расстройства

Растущее количество данных клинических исследований уже более 50 лет свидетельствует о том, что КД может способствовать улучшению течения ряда неврологических заболеваний, в частности у пациентов с лекарственно-устойчивой эпилепсией и эпилептическим статусом.

В метаанализе 2015 г. результатов 12 исследований (270 пациентов) применения различных вариантов КД получены данные о терапевтическом эффекте при лекарственно-устойчивой эпилепсии у 13–70% пациентов [25]. Аналогичные результаты были получены при анализе данных регистра в период с 1946 по 2019 гг., куда вошли 13 исследований (932 участника: 711 детей (от 4 мес до 18 лет) и 221 взрослый (от 16 лет и старше)) [26]. Авторы сделали вывод о наличии эффективности применения КД у детей с лекарственно-устойчивой эпилепсией, однако в связи с ограниченным количеством исследований и их небольшой выборкой, доказательств использования КД у взрослых недостаточно. Основываясь на данных наблюдательных исследований, большинство авторов считают, что классический протокол КД снижает проявления судорожного синдрома на ≥50% у 22–70% пациентов, комбинированный — на ≥50% у 12–67% пациентов, предполагая, что наиболее эффективным применение КД может быть у пациентов с генерализованной эпилепсией [27] (табл. 2).

**Table:** Таблица 2. Исследование эффективности применения КД у пациентов с лекарственно-устойчивой эпилепсией, включенных в метаанализ от 2015 г. [25]

Sirven J. и др., 2005 г.	8	32,2 (19–45)	11	4 Отмечена значительная неоднородность групп	Запор и нарушения менструального цикла	Субъективное улучшение концентрации внимания	↗ХС, ЛПВП, ТГ в сыворотке во время КД	Не описано	Терапевтический эффект у n=20%
Kossoff E.H. и др., 2008 г.	6	35,5 (18–53)	30	16	Нет	Нет	↗ХС, мочевины, отношения кальция к креатинину в моче	В среднем минус 6,8 кг	Через 1 и 3 мес у n=47% ↘приступов >50% Через 6 месяцев у n=33%
Carrette E. и др., 2008 г.	6	42 (30–54)	8	7	Нет	Нет	Не описаны	Не описано	↘приступов >50%, 1/3 >30% и 1/3 <30% у n=1 из n=3
Mosek A. и др., 2009 г.	3	28±6 (18–45)	9	2	Диарея (n=1)	Улучшение внимания и концентрации (n=3). Качество жизни не изменилось, отмечено снижение удовольствия от еды и более частое чувство голода.	↗ЛПВП в сыворотке во время КД	Не описано	Терапевтический эффект у 15% (n=2)
Klein P. и др., 2010 г.	26	42 (24–65)	12	6	Тошнота, рвота, диарея, запор	Нет	Не описано	Не описано	Через >4 мес ↘ количества приступов на 85% у 12%;
Smith М. и др., 2011 г.	12	36,5 (18–55)	18	4	через 3 мес КД новый тип приступа (судороги левой руки) (n=1)	Нет. Отмечены финансовые и материально-технические трудности, связанные с соблюдением диеты	↘ ТГ, ХС через год — 3,82–6,61 мм (норма <5,2 мм) со средним значением 5,24 мм	Не описано	Через 3 мес ↘ количества приступов более чем на 50% у 12%; через 6 мес — у 28%; через 12 мес — у 21%
Cervenka M.C. и др., 2012 г.	3	30 (18–66)	25	16	Диарея (n=2), гастроэзофагеальный рефлюкс (n=1), метеоризм (n=1), боль в животе (n=1), слабость (n=1), нарушения менструального цикла (n=1) и увеличение частота приступов (n=1)	Нет	Значительное ↗ХС, ЛПНП было отмечено у n=1	Не описано	Через 1 мес у n=9 (41%) ↘ приступов на 50% в том числе n=1 (5%) на >90% Через 3 мес у n=6 (27%) ↘ более чем на 50%, в том числе у n=3 (14%) более чем на >90% В целом у 41% участников частота приступов снизилась более чем на 50% через 1 мес и на 27% через 3 мес
Kossoff E.H. и др., 2013 г.	6	24,3 (15–44)	8	3	Не описаны	Нет. Отмечены трудности приверженности КД	Не описаны	Не описано	Через 1 мес у n=6 (75%) ↘ приступов на 50% Через 3 мес у n=5 (63%) улучшение на 50%
Nei M. и др., 2014 г.	24	32 (11–51)	28	16	Метаболический ацидоз (n=1), боли в животе (n=1), запоры	Нет. Отмечены трудности при соблюдении (n=11)	↗ХС, ТГ и соотношения ХС / ЛПВП, но ЛПВП оставались стабильными. ↘ селена, карнитина	Не описано	↘ частота приступов y n=15 (52%) ↘ снижение приступов на ≥50% У n=13 (45%), в том числе у n=6 (21%) на ≥80%. Не было улучшений или досрочного прекращения диеты у n=9 (31%). ↗частоты приступов на ≥50% у n=3

Интересен опыт использования КД в педиатрии. Недавний отчет показывает, что при сопутствующей неврологической патологии применение КД относительно безопасно и эффективно даже для младенцев в возрасте от 6 нед [28, 29]. Однако авторами настоятельно рекомендуется посетить лечащего врача для определения типа(ов) припадков и исключения метаболических нарушений, которые могут стать противопоказаниям для назначения КД. Результаты применения КД при эпилепсии подтвердили данные более ранних исследований о положительном терапевтическом эффекте. Так, метаанализ 7 исследований с участием 427 детей и подростков, больных эпилепсией, продемонстрировал достоверное снижение количества приступов в среднем до 85% через 3 мес соблюдения КД [29], но на фоне развития бОльшего количества побочных эффектов. Однако авторы подчеркивают, что ни в одном исследовании не оценивались качество жизни, степень изменения когнитивных или поведенческих функций на фоне КД. Изучение этого аспекта имеет важное значение, например, в исследованиях in vivo ученые Калифорнийского университета в 2021 г. получили данные о негативном влиянии КД на когнитивные способности мышей в условиях гипоксии [30]. При этом полученные результаты несколько противоречат метаанализу данных 24 исследований (n=1221), куда вошли и пациенты с БА и БП [31].

Дегенеративные болезни центральной нервной системы

Учитывая существенную роль дисфункции митохондрий и нарушения обмена углеводов в теориях патогенеза БА, можно предположить определенный терапевтический эффект КД и при подобной патологии.

За последние 15 лет опубликовано несколько работ, где авторы наблюдали благоприятное влияние КД на моторные и немоторные симптомы БП [31][32]. При БП есть ряд нерешенных вопросов, требующих дальнейшего изучения: предположение, что кетоны, усиливая митохондриальное окислительное фосфорилирование и стимулируя биогенез митохондрий, могут оказывать положительный терапевтический эффект на метаболизм центральных и периферических нейронов [32]; в противовес же выступают сведения, что на высокоуглеводном рационе наблюдается повышение уровня дофамина в головном мозге и спинномозговой жидкости. Так, на фоне КД в течение не менее 6–8 нед у 47 (исследование завершили 38) пациентов с БП было выявлено значительное улучшение памяти (p=0,03), двигательных и немоторных функций (p<0,001) [31][32].

В первом рандомизированном контролируемом исследовании применения КД у 20 больных БА было продемонстрировано улучшение краткосрочных когнитивных функций (проводилась оценка внимания, памяти, разговорной речи) [33]. Ряд исследователей предположили, что регулярный прием добавок кетонового моноэфира ((R)-3гидроксибутил(R)-3-гидроксибутирата) как альтернатива КД также может привести к повышению уровня β-НВ и схожим терапевтическим эффектам [34]. Однако обращает на себя внимание вывод ученых, что наиболее эффективным применение КД при БА было у пациентов без аллеля APOE ε4 [35].

Механизм терапевтического противосудорожного эффекта КД у пациентов до конца не изучен. Однако, согласно недавнему обзору [36], все большее количество экспериментальных исследований демонстрирует плейотропное противосудорожное и нейрозащитное действия кетонов, таких как [37]:

1.влияние на скорость высвобождения нейромедиаторов и полярности нервных мембран, приводящие к ослаблению повышенной возбудимости;

2.избыточное накопление γ-аминомасляной кислоты (ГАМК) в пресинаптических сосудах;

3.модуляция АТФ-чувствительных калиевых каналов;

4.избирательное подавление рецепторов α-амино-3-гидроксил-5-метил-4-изоксазолепропионовой кислоты (AMPA) за счет накопления жирных кислот;

5.улучшение функции митохондрий за счет увеличения запасов энергии в сочетании со снижением продукции активных форм кислорода (АФК):

6.геномные эффекты:

7.противовоспалительное и нейропротекторное свойство:

8.метаболическая регуляция:

Доказательств возникновения побочных эффектов лекарственной терапии неврологических заболеваний на фоне соблюдения КД мало. В литературе упоминается возможный риск развития ацидоза на фоне приема ингибиторов карбоангидразы (ацетазоламид, топирамат, зонисамид) в сочетании с КД [16][40].

Научные данные, собранные в результате клинических исследований последнего 15-летия, подтверждают эффективность применения КД при эпилепсии и БА у взрослых. Однако общая оценка всех возможных последствий КД остается ограниченной вследствие неоднородности групп наблюдения, небольших размеров выборки, а также применения разнообразных вариантов протоколов КД. Использование КД, возможно, будет полезно в качестве дополнительной терапии к фармакологическим и нефармакологическим подходам, с проведением тщательной оценки всех аргументов «за» и «против» (табл. 3).

**Table:** Таблица 3. Исследование эффективности применения КД у пациентов с патологией нервной системы *РКИ — рандомизированное клиническое исследование.

Автор	Длительность, мес	Средний возраст, лет	Количество участников, n	Побочные эффекты	Оценка качества жизни	Лабораторные данные	Снижение веса	Вывод авторов
Ye F. и др., 2015 г. [25]	2–8	42 (18–66)	270 Метаанализ 12 РКИ*	Диспептические явления	Не проводилась, приверженность КД в 45%	↗ХС, ЛПВП, ТГ во время КД	Не описано	Терапевтический эффекте при лекарственно-устойчивой эпилепсии у n=13–70%
Martin K. и др., 2016 г. [26]	3–6	4 мес–18 лет	932: 711 детей 221 взрослых. Метаанализ 13 РКИ	Диспептические явления (рвота, запор). Реже: диарея, дисфагия, инфекция нижних дыхательных путей, острый панкреати, снижение плотности костного матрикса, желчные камни, нефрокальциноз, гиперхолестеринемия, ацидоз, обезвоживание, тахикардия, гипогликемия, голод и боли в животе	Не описано	Не описано	Да	КД эффективна у детей с лекарственно-устойчивой эпилепсией, однако, в связи с ограниченным количеством исследований и их небольшой выборкой, доказательств использования КД у взрослых недостаточно.
Christensen M.G. и др., 2021 г. [31]	1–1.5	4	1221 Метаанализ 24 РКИ (n=21 — пациенты с эпилепсией, n=2 — с БА, n=1 — с БП)	Не описано	Улучшение памяти через 6 нед при БА (p =0,03)	Не описано	Не описано	↘ количества приступов по сравнению с исходным уровнем во всех исследованиях. На КД улучшение двигательных и немоторных функций при БП через 8 нед (p<0,001).

Эндокринная система

Ожирение

Сторонники КД считают, что принятая в США во второй половине ХХ в. парадигма питания с более высоким содержанием углеводов на фоне пониженной доли жира могла быть причиной увеличения распространенности ожирения, основного фактора риска СД 2 типа [41]. Более того, недавние исследования показывают, что метаболические эффекты продуктов питания больше, чем их калорийность, определяют вес в долгосрочной перспективе. Согласно углеводно-инсулиновой модели ожирения, переработанные углеводы (большая часть хлебобулочных и кондитерских изделий, продуктов на основе картофеля, риса), заменившие жиры в эпоху обезжиренной диеты, способствовали увеличению чувства голода и снижению расхода энергии, что приводило к отложению жира у предрасположенных к ожирению и СД лиц [42]. Интересно мнение, что кетоз, возможно, является одной из причин подавления аппетита при соблюдении КД [43]. В связи с чем КД часто применяются для потери или поддержания веса. Однако результаты клинических испытаний, изучающих влияние КД на аппетит, противоречивы, так же, как спорными остаются возможные последствия оказываемых метаболических эффектов данного рациона.

Ряд авторов считают, что результат применения КД у пациентов с избыточным весом может зависеть от секреции инсулина на старте протокола [44]. Открытым остается и важнейший вопрос снижения чувствительности к инсулину и формирования нарушения толерантности к глюкозе на фоне длительного соблюдения КД [44]. Проведенные исследования in vivo также продемонстрировали разные, а иногда и противоречивые результаты.

Еще один запрос, ассоциированный с метаболическим синдромом и возможностью его контроля за счет КД, связан со старением. Так, в известном исследовании Ямазаки T. (Yamazaki T.) и соавт. в моделях на мышах были получены данные об увеличении продолжительности их жизни при соблюдении КД [45][46]. Однако у испытуемых мышей динамики снижения веса не наблюдалось, более того, на фоне КД в ходе проведения перорального глюкозотолерантного теста через месяц КД регистрировались высокие показатели гликемии, а также фосфорилированного AS-160 (Akt-субстрат 160 кДа) в печени, известного как ключевой медиатор чувствительности к инсулину. Подтверждением снижения толерантности к глюкозе на КД можно считать и данные исследования in vivo под руководством Элленброек Дж. (Ellenbroek J.), в которых через 22 нед соблюдения КД регистрировалось уменьшение массы β-клеток поджелудочной железы на фоне стеатоза печени [47]. Было показано, что по завершении КД наблюдалось улучшение показателей обмена углеводов, причем в короткие сроки [48].

При сравнении эффективности КД, низкожировой и средиземноморской диеты в ходе исследования DIRECT, в которое были включены 322 пациента с ожирением (36 из них имели СД2), во всех вариантах питания наблюдались эффективное снижение массы тела, уменьшение объема талии и уровней провоспалительных маркеров, однако нормализация гликемии наблюдалась только на фоне средиземноморской диеты [49].

Сахарный диабет 2 типа

Известно, что до появления инсулинотерапии КД была одним из способов продления жизни больных СД 1 типа и лучшей компенсации СД 2 типа [50]. Открытие инсулина в 1920-х гг. позволило пациентам с СД придерживаться более разнообразного рациона. Однако, несмотря на наличие большого числа аналогов инсулина, пероральных сахароснижающих препаратов (ПССП) и способов лечения сопутствующих состояний, таких как дислипидемия, гипертония и коагулопатия, число лиц с осложнениями СД продолжает расти. Данные литературы о применении КД при СД 2 типа несколько противоречивы. Несмотря на наличие доказательств того, что сокращение потребления углеводов снижает массу тела и улучшает контроль глюкозы у больных СД 2 типа, данных об устойчивости, безопасности и эффективности в долгосрочной перспективе представлено мало.

В настоящее время ряд ассоциаций не рекомендует применение КД у лиц с нарушением углеводного обмена, в первую очередь по причине высокого риска развития гипогликемии и ассоциированных с этим состоянием последствий [18]. Существует мнение, что КТ могут стимулировать секрецию инсулина у здоровых людей [51]. При этом ассоциации диетологов и эндокринологов Канады, Австралии и Великобритании рассматривают возможность применения КД, однако длительностью не более 3 мес и с обязательным наблюдением лечащего врача [52]. Особое внимание стоит уделить пациентам на терапии ингибиторами SGLT-2, которые могут войти в группу высокого риска диабетического кетоацидоза при выборе КД, что является абсолютным противопоказанием [53]. Дозу инсулина, препаратов класса сульфонилмочевины и глинидов следует постепенно снижать (примерно на 50%); бигуаниды, ингибиторы дипептидилпептидазы-4 и агонисты глюкагоноподобного пептида-1 следует отменить.

В двух метаанализах, опубликованных в 2018 и 2019 гг., были изучены данные наблюдения 2412 и 2132 участников соответственно, которые придерживались диет с очень низким (<50 г в сутки) и низким содержанием углеводов (50–130 г в сутки) в течение 6 мес и продемонстрировали значимое снижение уровня гликированного гемоглобина (НbА1с) и массы тела [54][55]. Привлекают внимание данные наблюдения пациентов в течение года после 10 нед КД — у 26,2% (n=195) без сопутствующей ПССП наблюдалась нормализация показателей гликемии, у 40,4% (n=289) терапия была редуцирована [56]. Почти половина участников потеряли не менее 5% своего веса, а у пациентов с исходным уровнем HbA1c ≥7,5% наблюдалось снижение HbA1c с 9,2% до 7,1% (р<0,001). К сожалению, подобные позитивные результаты применить к более широкой практике затруднительно, так как относятся только к 528 (52,80%) пациентам, завершившим исследование. Похожие результаты были получены и в других исследованиях, но возникли те же вопросы — успешно завершить протокол смогли только половина и меньше пациентов, анализ возможных побочных эффектов проводился не в достаточном объеме и без участия контрольной группы [57, 58]. Следует отметить, что в большинстве случаев пациенты получали дополнительную поддержку (например, занятия с врачом лечебной физкультуры, консультирование исследователей, советы по гигиене сна, групповые занятия), что могло также способствовать положительному результату. Еще раз подчеркнем, что в большинстве подобных исследований в области питания разработать хорошо контролируемые исследования сложно. Отсутствие приверженности лечению у большинства, частый отказ от участия, ограничения возможности долгосрочного контроля гликемии и массы тела испытуемых вносят определенный вклад, в том числе, и в статистическую обработку данных. В метаанализе 153 исследований было отмечено — достигнутые положительные результаты КД со временем часто терялись, что указывает на возможную бесперспективность на средних и длительных периодах соблюдения [59], хотя подобный вывод можно применить к любому варианту питания. При этом низкую приверженность отметили и авторы метаанализа 14 исследований, причинами которой они считают отсутствие психологической поддержки пациентов [60]. Исследователи обратили внимание, что последствия влияния КД на обмен жиров неоднородны и лабораторные результаты часто диаметрально противоположны. Подчеркнуто положительное влияние КД на синтез провоспалительных маркеров и уровень HbA1c, однако данный эффект мог быть связан с ограничением калорий в целом [60].

Привлекают внимание результаты наблюдения 115 пациентов с ожирением и СД 2 типа в течение 52 нед, которые были случайным образом распределены на группы: 1) рацион с очень низким содержанием углеводов, высоким содержанием ненасыщенных и низким содержанием насыщенных жиров, 2) изокалорийная диета с высоким содержанием углеводов и низким содержанием жиров. В обоих случаях наблюдалось снижение массы тела и уровня HbA1c. В 1-й группе регистрировалось улучшение липидного профиля и снижение количества ПССП [61]. Те же авторы сообщили о долгосрочной устойчивости этих эффектов: через 2 года после рандомизации на фоне отсутствия неблагоприятных исходов [57], по сути, ремиссии СД 2 типа, что согласуется с данными других исследований [62][63]. Например, в открытом нерандомизированном контролируемом исследовании пациентов с СД 2 типа на КД в сравнении с привычным лечением. Через 1 год у пациентов на КД наблюдался схожий с подобными исследованиями результат (снижение уровня НbА1c, значительное улучшение признаков неалкогольной жировой болезни печени), однако наблюдалось повышение уровня липопротеидов низкой плотности (ЛПНП) [64], что вызывает определенную обеспокоенность относительно сердечно-сосудистых рисков.

Сахарный диабет 1 типа

Существует мнение, что увеличение численности рода Bacteroides негативно влияет на микробиоту кишечника, что может отразиться на состоянии/функции β-клеток [65]. В связи с этим ряд авторов предположили возможное позитивное влияние КД при СД 1 типа. Канадская ассоциация изучения диабета (Diabetes Canada) приводит данные 2 небольших исследований (n=20) пациентов, находящихся на инсулинотерапии и КД [66]. Авторы сообщают о достоверном снижении НbА1c, однако контроль уровня артериального давления, динамики креатинина или липидного профиля, непрерывного мониторинга глюкозы (CGM) или качества жизни не проводился. Стоит выделить, что до сих пор неизвестны отдаленные результаты применения КД у пациентов с СД 1 типа, особенно у детей и подростков [67]. Более того, Де Бок (de Bock) и соавт. описывают ряд случаев формирования антропометрического дефицита и более высокого метаболического профиля сердечно-сосудистого риска и утомляемости у детей с СД 1 типа на фоне ограничения потребления углеводов [68] (табл. 4).

**Table:** Таблица 4. Исследование эффективности применения КД у пациентов с патологией эндокринной системы *РКИ — рандомизированное клиническое исследование.

Shai I. и др., 2008 г. Yokose C. и др. (второй пересмотр результатов), 2020 г. [49]	24	52	322 Случайным образом разделены на 3 группы:низкокалорийная диета с низким уровнем жиров (1-я группа),средиземноморская (2-я группа),КД (3-я группа)	Не описано	Не проводилась. Авторами отмечена наименьшая приверженность (78%) в 3-й группе по сравнению с 90,45% в 1-й и 85,3% в 3-й	Относительное ↘ отношения ХС ЛПВП — в 3-й группе 20%,в 1-й группе — 12% (р=0,01). ↘ ТГ на КД. ↗адипонектина и ↘ лептина во всех группах,↘ СРБ во 2-й и 3-й группах. ↘ уровня глюкозы среди больных СД (n=36) наблюдалось только во 2-й группе	1-я группа — 2,9 кг, 2-я группа — 4,4 кг, 3-я группа — 4,7 кг (р<0,001)	При сравнении эффективности 2 и 3-й групп, в обоих вариантах питания наблюдалось эффективное снижение массы тела, однако нормализация гликемии наблюдалась только во 2-й группе
Sainsbury E. и др., 2018 г. [54]	Анализ данных за период с 1.01.1980 г. по 31.08.2016 г.	4	2412 25 РКИ	Не описано	Не описано	Снижение HbA1c через 3 мес (95% ДИ -0,71–-0,23) и через 6 мес (95% ДИ -0,62–-0,09), без существенной разницы через 12 или 24 мес Краткосрочные результаты (3–6 мес) указывают либо на отсутствие изменений, либо на небольшое снижение ↘ ХС и ЛПНП как на диетах с ограничением углеводов, так и на диетах с высоким содержанием углеводов. В 9 из 20 исследований сообщалось о большем ↗ ЛПВП	Через 3 мес большая потеря веса при ограничении углеводов (<26% от общей калорийности) (95% ДИ: -1,93, -0,23, n=12 исследований), на 2,47 кг (95% ДИ: -3,33–-1,60)	Применение КД в краткосрочной перспективе (3–6 мес) приводит к большему снижению HbA1c, чем диеты с высоким содержанием углеводов при СД2
Mcardle P.D. и др., 2018 г. [55]	Анализ данных за период с 1976 г. по апрель 2018 г.	4	2132 1402 РКИ (25 РКИ соответствовали критериям включения)	Не описано	Не проводилась. Отмечены разные определения понятий низкого содержания углеводов в разных исследованиях	Отсутствие влияния на HbA1c ограничения количества углеводов. (95% ДИ –0,27, 0,08 (р=0,30))	Не описано	Анализ подгрупп диет, содержащих 50–130 г углеводов, привел к выводу о значимом влиянии на HbA1c в пользу применения КД продолжительностью ≤6 мес (95% ДИ –0,75, –0,23 (р<0,001))
Tinguely D. и др., 2021 г. [60]	7	46,4	1262 14 РКИ ИМТ более 30 кг/м2	Легкие побочные эффекты: головные боли, тошнота и рвота (у 80% пациентов на КД по сравнению с 41% контрольной популяции (низкокалорийная диета)) (р<0,001). В конце исследования — запор и ортостатическая гипотензия	Не проводилась. Отмечена низкая приверженность	В краткосрочных исследованиях улучшение HbA1c было различным:↘на 0,6% через 3 нед до 0,9% через 4 мес, на 1,3% через 32 нед (p=0,02), с 8,9 до 5,6% (p<0,0001) через 90 дней. Влияние на обмен жиров неоднородно, диаметрально противоположные результаты исследований	Изменения веса зависят от продолжительности интервенционных испытаний. До 6 мес соблюдения потеря в весе 3,7 кг, p=0,026 без сохранения эффекта к 12-му месяцу Только в 1 РКИ из 14 проводился анализ состава тела	Достигнутые положительные результаты КД со временем часто терялись, что указывает на возможную бесперспективность на средних и длительных периодах соблюдения

Анализ литературных данных подтверждает положительный эффект применения КД на снижение массы тела, улучшение гликемического профиля при наличии СД 2 типа. Однако практически все исследователи подчеркивают необходимость объективности и индивидуального подхода при снижении потребления углеводов, соответствующих КД, также выделяют важность постоянного контроля липидного профиля, своевременной коррекции сахароснижающей терапии и строгих сроков применения КД (максимум до 10–12 нед) в связи с отсутствием данных в отсроченном периоде и наличием высокого сердечно-сосудистого риска при СД. Вопрос применения КД при СД 1 типа остается открытым в связи с малой выборкой пациентов и не рекомендован большинством ассоциаций. Необходимы дальнейшие крупномасштабные долгосрочные хорошо спланированные рандомизированные исследования для оценки безопасности и эффективности КД (табл. 5).

**Table:** Таблица 5. Исследование эффективности применения КД у пациентов с СД *РКИ — рандомизированное клиническое исследование, АСТ — аспартатаминотрансфераза, АЛТ — аланинаминотрансфераза, ТЗ — тиазолидиндионы, МЕТ — метформин, СМ — сульфонилмочевина, ССС — сердечно-сосудистые события, ИМТ — индекс массы тела, ХПН — хроническая почечная недостаточность, НЖ — низкожировая диета; ДПП-4 — дипептидилпептидаза-4, НКД — низкокалорийная диета.

Wong K. и др., 2020 г.	6–19	54,5	14 (СД2=11, СД1=3) ИМТ 31,5±5,1 кг/м2	Нет	Не описано	Улучшение гликемического контроля	Нет	Да	КД может быть использована при СД при условии постоянного медицинского контроля
Webster C.C. и др., 2019 г.	7–11	57±10	СД2 (n=28) Длительность СД2 7.4 г. (3,5–13,2) ИМТ 30±6 кг/м2	Эпизоды гипогликемии	Не описано	HbA1c 7,5% (6,5–9,5%) до начала КД, HbA1c 5,8 (5,4–6,2)% после	Нет	Да 16 (7–31) кг	КД может быть использована при СД при условии постоянного медицинского контроля
Walton C.M. и др., 2019 г.	3	38, 3±2,6	СД2 (n=11) ИМТ 36,3±1,4 кг/м2	Не описано	Не описано	HbA1c 8,9±0,4% до начала КД. ↘ HbA1с (p<0.0001), ↗ ЛПВП (с 43,1 до 52,3±3,3 мг/дл (p<0,005), ↘ ТГ (с 177±19,8 до 92,1± 8,7 мг/дл (p<0,005),	Нет	Да В среднем на 8,4±0,4 кг	КД может быть использована при СД, является альтернативой терапии ПССП (стимуляторы секреции инсулина)
Romano L. и др., 2019 г.	2	56±9,72	СД 2 (n=20). Длительность СД2 5,9±1,7 года. Терапия: нет (n=8), МЕТ (n=15), СМ (n=5), инсулин (10) ИМТ 37,1±6,8 кг/м2	Нет	Нет	HbA1с 7,3±1,1% до начала КД, ↘ HbA1с 6,16±0,07 после КД, HOMA-индекс до начала КД 7,47±2,07, после ↘ 2,13±0,88, ↘ AСT (36,75±5,06 до начала КД, после 21,21±4,49) ↘ AЛT (45,08±6,97 до начала КД, после 24,07±5,69)	Да. Плотность костной ткани (ДХА) не изменилась. Уменьшение жировой ткани на 17,75% (8,1 кг в среднем)	Да В среднем на 11,5 ±8,7 кг через 4 нед, через 8 нед на 15,36 ±7,3 кг. ИМТ через 4 нед 34,75±6,50 кг/м2, через 8 нед 33,25±5,99 кг/м2	КД может быть использована при СД, необходимы дальнейшие исследования
Barbosa-Yañez R.L. и др., 2018 г.	3 нед	63 (42–76)	СД2 (n=36). ИМТ 35,0±5,0 кг/м2. Случайно разделены на 2 группы: в 1-й — КД (n=18), во 2-й — НЖ (n=18)	Не описано	Нет	HbA1c8,9±0,4 % до КД, после 6,7% в 1-й группе, 6,1% во 2-й. ОХС200 мг/дл в 1-й группе, 173 мг/дл во 2-й. ЛПНП127 мг/дл в 1-й группе,106 мг/дл во 2-й.	Да. Висцеральный жир меньше на 0,4 кг, жировая масса на 1,7 кг	Да В среднем на 4,1 кг	Оба варианта: КД и НЖ способствовали снижению веса, уровня липидов и жировой массы. Пациенты 2-й группы продемонстрировали более благоприятное влияние НЖ на состояние эндотелия сосудов
Hallberg S.J. и др., 2018 г.	12	53,75±8,35	СД2(n=349,262 на терапии). Исследование завершили 83%. ИМТ 40,4±8,8 кг/м2. Длительность СД2 8,44±7,22%. ПССП: МЕТ (n=15), СМ (n=62), инсулин (10)	У n=1 (0,38% от начавших) — клинически значимое повышение уровня креатинина в сыворотке. Субклинический гипотиреоз (n=2). CCC (n=3), метастатическая нейроэндокринная карцинома (n=1), рак легких с множественными поражениями головного мозга (n=1), смерть в исходе ХПН (n=1), гиперкалиемия (n=1). Эпизод гипогликемии (n=1)	Не описано	↘ HbA1c 7,6±1,5 до КД, после до 6,3±0,07%, ↘ HOMA-IR — 55%, ↗ ЛПНП + 10%, ↘ креатинина сыворотки и ферментов печени (АЛТ, АСТ и ЩФ). Аполипопротеин B без изменений. Среднее значение мочевой кислоты временно повысилось через 70 дней, но не изменилось через 1 год	Не описано	Да. В среднем на 13,8±0,71 кг	Количество пациентов, получающих ПССП, снизилось с 56,9±3,1% до 29,7±3,0%. У 94% пользователей инсулинотерапия была скорректирована или отменена; препараты СМ полностью отменены. Данная модель питания может помочь пациентам с СД 2 типа скорректировать обмен углеводов, снизить вес
Saslow L.R. и др., 2017 г.	12	Старше 18	СД2 (n=34). ИМТ >25 кг/м2. Случайно разделены на 2 группы: КД (n=16) и НЖ (n=18)	Не описано	Нет 85,3% завершили исследование	HbA1cв 1-й группедо КД 6,6%, через год 6,1%, во 2-й группедо НЖ 6,9%,через год 6,7%. ТГв 1-й группедо КД 102,6 мг/дл,через год 92,7 мг/дл,во 2-й группе до НЖ 158,9 мг/дл,через год 173,4 мг/дл. ЛПНПв 1-й группедо КД 88,7 мг/дл,через год 95,6 мг/дл, во 2-й группе до НЖ 98,1 мг/дл,через год 96,1 мг/дл	Нет	Да Вес в 1-й группе снизился на 7,9 кг, во 2-й группе — на 1,7 кг	Коррекция терапии в 1-й группе проводилась чаще (6/10 прекратили прием препаратов). Результаты показывают, что взрослые с предиабетом или инсулинозависимым СД 2 типа могут улучшить гликемический контроль с меньшим количеством лекарств на фоне КД
Wycherley T.P. и др., 2016 г.	12	58,5±1,0	СД2 (n=11). ИМТ 34,6±0,4 кг/м2. Случайно разделены на 2 группы: КД (n=58) и изокалорийная диета (n=57)	Не описано	Нет. n=78 завершили исследование	HbA1cв 1-й группедо КД 7,26±0,14%, через год 6,26±0,12%, во 2-й группе до НЖ 7,42±0,15%, через год 6,33±0,13%	Нет	Да. Вес в 1-й группе снизился на 10,2 ±0,1 кг, во 2-й группе — на 10,9 ±0,2 кг	В обеих группах наблюдалось одинаковое снижение веса и HbA1c. Предположительно, КД не оказывает отрицательного воздействия на функцию эндотелия
Goday А. и др., 2016 г.	4	54,53 (30–65)	СД2 (n=89). 80% получали терапию, длительность СД2 более 10 лет. ИМТ 33,1±1,6 кг/м2. Случайно разделены на 2 группы: КД (n=45) и НКД (n=44)	В группе КД астения (n=7), головная боль (n=9), тошнота (n=9), рвота (n=7), запор (n=2)	Нет	HbA1cв 1-й группе 6,9% до КД,через 4 мес 6,0%,во 2-й группе 6,8%,через 4 мес 6,4%. ЛПНПв 1-й группедо КД 112,7 мг/дл,через 4 мес 110,6 мг/дл, во 2-й группедо КД 109,8 мг/дл,через 4 мес 107,1 мг/дл. Между двумя исследуемыми группами не было обнаружено значительных различий в лабораторных параметрах безопасности	Не описано	Да. Вес в 1-й группе снизился на 14,7 кг, во 2-й группе — на 5,05 кг	КД наиболее эффективна для снижения массы тела и улучшения гликемического контроля, чем стандартная гипокалорийная диета, с безопасностью и хорошей переносимостью для пациентов с СД2
Tay J. и др., 2015 г.	12	58±7	СД2 (n=115). Длительность СД2 8±6 лет. ПССП: 1-я группа: ТЗ (n=5), МЕТ (n=79), СМ (n=20), инсулин (n=10), ГПП-1(n=2), ДПП-4 (n=2). 2-я группа: ТЗ (n=5), МЕТ (n=72), СМ (n=28), инсулин (n=11), ГПП-1(n=2), ДПП-4 (n=4). ИМТ 34,6±4,3 кг/м2. Случайно разделены на 2 группы: КД (n=57) и НКД (n=58)	21 участник (1-я группа (n=8);2-я группа (n=13)) сообщил о заболеваниях опорно-двигательного аппарата. 3 участника (1-я группа (n=2),2-я группа (n=1)) сообщили о желудочно-кишечных расстройствах (запоры и дивертикулит). Эпизод гипогликемии (1-я группа (n=1)). Нарушения сердечного ритма (2-я группа (n=1))	Нет. 68% завершили исследование	HbA1cв 1-й группе 7,3% до КД, через год 6,3%, во 2-й группе 7,4%, через год 6,4%. ЛПНПв 1-й группедо КД 2,5 ммоль/л, через год 2,4 ммоль/л, во 2-й группе до 2,4 ммоль/л, через год 2,2 ммоль/л. ТГв 1-й группедо КД 1,6 ммоль/л, через год 1,2 ммоль/л, во 2-й группе до 1,4 ммоль/л, через год 1,3 ммоль/л. В 1-й группе коррекция ПССП у 52% по сравнению с участниками 2-й группы (21%)		Да. ИМТ в 1-й группе снизился на 3,2 кг/м2, во 2-й группе — на 3,5 кг/м2. Потеря жировой ткани в 1-й группе составила 7,9 кг, во 2-й группе — 8,6 кг	Обе диеты позволили значительно снизить вес и уровень HbA1c и глюкозы натощак. Соблюдение КД ассоциировано с улучшением липидного и гликемического профиля, редуцирования ПССП
Hussain T.A. и др., 2012 г.	6	Старше 18 лет	Ожирение (n=363), из них СД2 n=102. ИМТ 37,3±0,3 кг/м2. Случайно разделены на 2 группы: КД (n=143) и НКД (n=220)	Не описано	Не описано	HbA1c 7,9% в среднем у пациентов с СД2 до старта диет. У пациентов с СД2 на фоне КД наблюдалось: ↘ общего холестерина (p<0,0001), ↘ ЛПНП (p<0,0001), ↗ ЛПВП (p<0,0001)	Не описано	Да, в обеих группах у пациентов с СД2	КД улучшает показатели гликемии. Необходим строгий медицинский контроль при соблюдении КД.

Репродуктивная система

Синдром поликистозных яичников

СПКЯ считается наиболее частым эндокринным заболеванием у женщин репродуктивного возраста с предполагаемой распространенностью от 6 до 15%, в зависимости от используемых диагностических критериев. Инсулинорезистентность играет ключевую роль в развитии СПКЯ. Исследований о применении КД в данной когорте пациентов немного [69][70]. Известны данные о 12-недельном наблюдении 14 женщин с избыточным весом и СПКЯ [71]. Проводились измерения состава тела, контроль биохимических и гормональных параметров крови, а также показателя Ферримана–Галлвея. Авторы сообщили о значительном снижении массы тела (в среднем -9,43 кг за 4 мес) преимущественно за счет висцеральной жировой ткани (в среднем 8,29 кг). Наблюдалось значительное снижение HOMA, отношение гонадотропинов, ТГ, общего холестерина и ЛПНП наряду с повышением уровней липопротеидов высокой плотности (ЛПВП). Схожие результаты были получены у 18 пациенток в китайской популяции при сравнении с контрольной группой [72]. Однако оценка показателей углеводного обмена, как и влияние КД на состояние пациенток с олигоменореей и бесплодием, не проводилась.

Синтез тестостерона

При анализе результатов наблюдения 40 мужчин с ИМТ более 25 кг/м2 в возрасте 45,8±2,42 года в среднем, соблюдавших VLCKD не менее 8 нед (в среднем 13,5±0,83 нед), было отмечено снижение веса на 21,05±1,44 кг; значительное улучшение показателей обмена углеводов и жиров; повышение уровня витамина D, общего тестостерона и лютеинизирующего гормона [73][74]. Однако оценка состава тела в динамике, как и сравнение с группой контроля, авторами не проводились.

Любопытны результаты эксперимента in vivo при добавлении КД к терапии тестостерона пропионатом у крыс-самцов с доброкачественной гиперплазией предстательной железы (ДГПЖ). Авторы отметили уменьшение объема железы и предположили перспективность КД уже у мужчин с ДГПЖ [75].

Несмотря на отсутствие данных о возможных последствиях длительного использования КД, все чаще подобный рацион выбирают с целью снизить или удержать вес лица с нормальной массой тела на фоне видимого благополучия. Так, у здоровых спортсменов (n=25) применение КД в течение 11 нед привело к повышению уровня тестостерона на фоне значимого повышения уровня ТГ при сравнении с группой здоровых добровольцев на западной диете [76]. Согласно метаанализу баз данных (13 исследований, 243 участника) от 2021 г., при сравнении эффектов различных диет на массу и состав тела в физически активных группах населения было выявлено более значимое снижение массы тела при соблюдении КД (2,7 кг, из которых 2,3 кг жировой ткани) [77]. Однако авторы ссылаются на возможный высокий риск систематической ошибки данного обзора, что требует дальнейшего изучения (табл. 6).

**Table:** Таблица 6. Исследование эффективности применения КД у пациентов с патологией репродуктивной системы

Bueno N.B. и др., 2013 г. [69]		Не описано	n=1577 (n=787 вошли в 1-ю группу НЖ, n=790 вошли во 2-ю группу КД). Средний ИМТ более 27,5 кг/м2 13 РКИ (5 РКИ соответствовало критериям включения)	Не описано	Не описано	Пациенты 2-й группы достигли значительно большего ↗ЛПНП (95% ДИ 0,04–0,2) ммоль/л, р=0,002). Оценка показателей углеводного обмена не дала статистически значимых результатов	Не описано	Пациенты 2-й группы достигли значительно большей потери веса: 0,91 кг (95% ДИ1,65, -0,17) р=0,02)	Пациенты на КД достигают большего снижения массы тела, объема талии, но они также демонстрируют большее увеличение уровней ЛПНП в течение последующих 12 мес или больше
Paoli A. и др., 2017 г. [71]	3	27,5±8,3	n=16 (завершили 14). ИМТ≥25 кг/м2	Не описано	Не описано. Влияние КД на другие исходы, такие как олигоменорея и бесплодие, не оценивалось	ЛГ в плазме (до КД 10,24±1,43 МЕ/млпосле КД 6,41±1,46 МЕ/мл; p<0,0001),общего тестостерона(до КД 47,43±6,08 нг/длпосле КД 40,71±5,77 нг/дл; p<0,0001),свободного тестостерона(до КД 0,96±0,60 пг/млпосле КД 0,56±0,30 пг/мл; p<0,0009),DHEAS (до КД 2,13±0,26 мкг/мл,после КД 1,70±0,20 мкг/мл; p<0,0001). Оценка показателей углеводного обмена не проводилась	Значительное снижение жировой массы (8,29 кг)	Снижение массы тела (-9,43 кг), ИМТ (-3,35)	КД может быть рассмотрена у пациенток с СПКЯ как эффективный метод снижения веса
Mongioì L.M. и др., 2016 г. [73]	3,2	45,8±2,42 года	n=40 (39 завершили). СД (n=3,7,5%), НТГ (n=11,27,5%). Инсулинорезистентность (n=32,80%) (индекс HOMA >2,5). ИМТ 37,5±1,1 кг/м2	Мочекаменная болезнь (n=1)	Не описано	Нормализация показателей углеводного обмена, ЛПНП, ТГ, печеночных ферментов (p<0,01; p<0,05). ↗ ЛПНП (n=5, 12,5%). Уровни креатинина в сыворотке немного ↗, но в пределах нормы со средним значением 0,84±0,02 мг/дл, мочевая кислота незначительно ↘. ↗ витамина D, ЛГ и тестостерона (р<0,01), ↘ ПСА (р<0,01) Уровень ГСПГ не исследовали	Не описано	Средняя потеря веса 21,05±1,44 кг	КД может быть эффективна у пациентов с ожирением

Обнадеживающие результаты о нормализации синтеза половых гормонов за счет потери жировой ткани на фоне КД требуют осторожного внимания, поскольку в большинство исследований включены небольшое количество пациентов и отсутствует группа контроля.

Сердечно-сосудистая система

Наибольший интерес, безусловно, вызывает влияние КД на риски развития сердечно-сосудистых заболеваний (CCЗ), поскольку в ряде исследований был отмечен рост уровня ЛПНП, что может способствовать более быстрому развитию атеросклероза. При этом на КД наблюдается снижение ТГ, а концентрация ЛПВП в плазме остается неизменной [74]. К. Реттерстол (K. Retterstol) и соавт. показали, что подобные изменения липидограммы отмечаются в том числе и у здоровых лиц с нормальным ИМТ на фоне соблюдения КД: в течение 3 нед уровень ЛПНП увеличился на 44% [78].

Сторонники КД утверждают, что увеличение доли жира в рационе на фоне снижения потребления углеводов приводит к росту количества крупных частиц ЛПНП в плазме, а не мелких и плотных, которые оказывают наибольший негативный эффект на состояние сосудистой стенки. Подобное предположение многие считают спорным, поскольку достоверные доказательства того, что размер частиц ЛПНП имеет решающее значение в формировании атеросклеротической бляшки, отсутствуют [79][80]. В исследовании MESA, посвященном патогенезу атеросклероза, малые и большие частицы ЛПНП были в равной степени связаны с увеличением толщины интима-медиа сонной артерии [81]. Более того, существует гипотеза, что небольшие плотные ЛПНП могут даже защищать от повторных сердечно-сосудистых событий [82].

Ранее считалось, что повышение ЛПНП на КД носит временный характер, и достаточно назначения статинов в случае, если ЛПНП не нормализуется после возвращения к стандартному сбалансированному рациону. Однако в сентябре 2019 г. Европейским сообществом кардиологов были опубликованы данные исследования NHANES (National Health and Nutrition Examination Survey), которое длилось 11 лет (1999–2010) и впервые позволило оценить отдаленные последствия влияния разнообразных вариантов питания на развитие ССЗ [83]. В исследовании участвовали 24 825 человек; 51% женщин и 49% мужчин; средний период наблюдения составил 6,4 года, средний возраст — 47,6 года. Получены следующие результаты: в группе пациентов на VLCKD и высокобелковом рационе был зарегистрирован самый высокий риск общей (32%) смертности, по причине ССЗ (50%), а также цереброваскулярной (51%) и онкопатологии (36%). При этом приверженцы VLCKD без ожирения (48%) умирали чаще, чем участники с ожирением (19%), что, безусловно, требует дальнейшего наблюдения, изучения и поиска возможных причин. Кроме того, результаты объединенных данных 9 проспективных когортных исследований, куда вошли 462 934 участника (средний период наблюдения 16,1 года), подтвердили наличие положительной связи между применением VLCKD и общей смертностью (относительный риск — ОР 1,22; 95% доверительный интервал — ДИ 1,06–1,39; р<0,001; I2=8,6), смертностью от ССЗ (ОР 1,13; 95% ДИ 1,02–1,24; р<0,001; I2=11,2), а также от онкопатологии (ОР 1,08; 95% ДИ 1,01–1,14; р=0,02; I2=10,3) [83]. В 2 когортных проспективных (срок наблюдения 26 лет) исследованиях последствий влияния КД в зависимости от пола (n=85 168 женщин, n=44 548 мужчин) были получены аналогичные данные (ОР 1,12; 95% ДИ 1,01–1,24; р=0,136) [84]. Японское проспективное когортное исследование последствий применения различных вариантов питания у 43 008 мужчин и 50 646 женщин в возрасте 45–75 лет через 17 лет наблюдения продемонстрировало высокий риск общей смертности на фоне КД и высокобелкового рациона. При этом питание с высоким содержанием белков и жиров растительного происхождения было связано с более низким риском общей смертности и смертности от ССЗ [85]. В систематическом обзоре 1 рандомизированного клинического испытания и 152 наблюдательных исследований моделей питания и общей смертности, включивших в себя 6 550 664 участников со средним сроком наблюдения 19 лет, был сделан вывод, что увеличение потребления овощей, фруктов, бобовых, орехов, цельного зерна, ненасыщенных растительных масел, рыбы и нежирного мяса или птицы связано со снижением риска общей смертности [86]. Авторы обратили внимание на важность проведения дополнительных исследований, где будут учтены, в том числе, и все особенности национальной кухни, а также образ жизни испытуемых.

Эффективное снижение жировой ткани на фоне патологического изменения липидного профиля при соблюдении КД может приводить к увеличению общей смертности, в том числе в исходе сердечно-сосудистых катастроф, что было продемонстрировано на большой когорте пациентов (табл. 7).

**Table:** Таблица 7. Исследование влияния КД на работу сердечно-сосудистой системы

Mazidi M. и др., 2019 г. [83]	11	47,6	24 825. СД 7,3–11,4%. ИМТ 28,4 кг/м2. Случайным образом разделены на группы с разным содержанием БЖУ, г/сут: Группа Q1 (n =6206). УВ: 367 (66%), Б: 77, Ж: 73. Группа Q2 (n =6205). УВ: 245 (57%), Б: 69, Ж: 65. Группа Q3 (n =6206). УВ: 205 (49%), Б: 72, Ж: 70. Группа Q4 (n =6209). УВ: 214 (39%), Б: 103, Ж: 105	432 случая смерти, в том числе 827 случаев смерти от рака, 709 случаев смерти от ССЗ и 228 случаев смерти т цереброваскулярных заболеваний	В группе приверженцев VLCKD и высокобелкового рациона был зарегистрирован самый высокий риск общей (32%) смертности, по причине ССЗ (50%), а также цереброваскулярной и (51%) и онкопатологии (36%). При этом приверженцы VLCKD без ожирения (48%) умирали чаще, чем участники с ожирением (19%), что, безусловно, требует дальнейшего наблюдения, изучения и поиска возможных причин
Mazidi M. и др., 2019 г. [83]	16,1 (4,9–29)	30–75	462 934 9 РКИ	Обнаружена значимая положительная связь между КД и общей смертностью (ОР 1,22; 1,06–1,39; р<0,001; n=8 исследований),смертностью от ССЗ(ОР 1,13; 95% ДИ 1,02–1,24; р<0,001; I2=11,2),а также от онкопатологии(ОР 1,08; 95% ДИ 1,01–1,14; р=0,02; I2=10,3)
Fung T.T. и др., 2010 г. [84]	26	34–59 женщины, 40–75 мужчины	129 716 без заболеваний ССС, рака или СД. n=85 168 женщин n=44 548 мужчин	12 555 смертей (2458 сердечно-сосудистых и 5780 связанных с раком) у женщин и 8678 смертей (2746 сердечно-сосудистых и 2960 связанных с раком) у мужчин	КД на основе животных жиров была связана с более высокой общей смертностью как у мужчин, так и у женщин, тогда как КД на основе овощей была связана с более низкими показателями общей смертности и в исходе сердечно-сосудистых заболеваний
Akter S. и др., 2021 г. [85]	16,9	56 (45–75)	93 654, из них СД2 у 4,7%. Наблюдение пациентов с помощью анкетирования и разделения на 11 категорий в соответствии с процентным содержанием энергии из углеводов, белков или жиров. ИМТ 23,5±0,02 кг/м2	13179 смертей (5246 связаны с раком, 5261 в исходе ССЗ и 1358 цереброваскулярных заболеваний)	КД с высоким содержанием животного белка и жира и диета с высоким содержанием углеводов и низким содержанием животного белка и жира были связаны с более высоким риском смертности. Между тем КД с высоким содержанием белков и жиров растительного происхождения была связана с более низким риском общей смертности и смертности от сердечно-сосудистых заболеваний
English L.K. и др., 2021 г. [86]	19	17–84	6 550 664. 28 стран. 153 РКИ — 1 рандомизированное исследование, 152 — наблюдательные (161–451 256 участников)	Общее количество смертей варьировалось от n=53 за 4 года наблюдения до n=51 702 за 13–18 лет наблюдения	Режимы питания с высоким содержанием питательных веществ, которые характеризовались повышенным потреблением овощей, фруктов, бобовых, орехов, цельного зерна, ненасыщенных растительных масел, рыбы и нежирного мяса или птицы, связаны со снижением риска общей смертности. Эти режимы питания включали относительно низкое потребление красного и переработанного мяса, жирных молочных продуктов и рафинированных углеводов или сладостей

Опорно-двигательный аппарат

Гипокальциемия и минеральная плотность костной ткани

Доказательств того, что низкоуглеводная диета оказывает отрицательное влияние как на минеральное содержание костной ткани (BMC), так и на минеральную плотность костной ткани (BMD), в частности в шейке бедренной кости и большом вертеле, и что этот эффект пропорционален степени снижения массы тела за счет как жировой, так и мышечной массы, мало. Биелогубы M. (Bielohuby M.) и соавт. в исследовании на крысах показали, что на фоне КД у животных отмечались снижение плотности костной массы и ухудшение механических свойств кости (предположительно, за счет уменьшения уровня циркулирующего инсулиноподобного фактора роста 1 (ИФР-1)) [87]. Однако следует заметить, что в эксперименте участвовали молодые животные, которые находились на КД в течение 4 нед, что по аналогии с человеческими мерками является очень долгим периодом времени. Ранее были опубликованы данные, что общий и ионизированный кальций, а также паратиреоидный гормон и кальцитонин остаются стабильными в течение месяца соблюдения КД. Однако при более длительном соблюдении КД, например, у детей с эпилепсией, наблюдается прогрессивное снижение минеральной плотности костной ткани [88]. В связи с чем для профилактики переломов в будущем рекомендуется обеспечить адекватное потребление кальция, компенсировать дефицит витамина D, а также до старта КД определить, находится ли пациент в группе риска.

Гиперурикемия

Отмечено, что на фоне ограничения потребления углеводов и снижения калорийности пищи менее 900 ккал/день уровень мочевой кислоты в сыворотке крови увеличивается. Приводятся данные, что гиперурикемия достигает пика через 1–2 нед КД, а затем чаще всего снижается, возвращаясь к исходному уровню [49]. Это наблюдение необходимо учитывать у пациентов с наличием подагры в анамнезе, что позволит заранее разработать меры профилактики и провести необходимую коррекцию терапии. Тем не менее были описаны приступы острого подагрического артрита на фоне КД, хоть и менее чем у 1% пациентов [89].

При наличии сопутствующей патологии кальций-фосфорного обмена, а также высоких значений мочевой кислоты в крови при назначении КД необходимы контроль дефицитов микроэлементов и коррекция сопутствующей терапии.

Желудочно-кишечный тракт

Галитоз, тошнота и обстипация

Присутствие КТ в крови и их выведение с мочой вызывают кетонемию и кетонурию. Ацетон (вырабатываемый спонтанным декарбоксилированием ацетоацетата), являясь очень летучим соединением, выводится в основном через легкие, вызывая появление характерного «фруктового запаха» изо рта. Подобное состояние относят к галитозам и считают одним из симптомов кетоза, который может быть физиологическим (голодание, низкоуглеводная диета, посттренировочный период) или иметь патологический характер [25].

Тошнота и рвота в большинстве случаев носят временный характер. Предполагают, что высокое содержание жиров при КД увеличивает время опорожнения желудка и тем самым способствует развитию гастроэзофагеальной рефлюксной болезни, тошноты и рвоты. При появлении подобных жалоб рекомендуется частое дробное питание, консультация гастроэнтеролога с решением вопроса о назначении соответствующей терапии. Причинами запора могут быть снижение потребления клетчатки и/или уменьшение объема потребляемой пищи [26]. Поэтому у пациентов с отягощенным анамнезом, наличием хронического запора, дивертикулита или геморроя необходимо рассмотреть возможность дополнительного введения пищевых волокон еще до старта КД. Также в литературе описаны случаи развития острого панкреатита и гепатита, которые на фоне КД встречаются редко, но в сочетании с применением противоэпилептических препаратов, в том числе, с низкой вероятностью, но могут привести к летальному исходу [5].

Желчнокаменная болезнь

Предполагается, что в основе формирования холестериновых конкрементов в желчном пузыре лежит перенасыщение желчи холестерином, а это, в свою очередь, приводит к его кристаллизации и последующему камнеобразованию. Преобладание жиров в рационе является фактором риска развития калькулезного холецистита. Характерное для этого состояния снижение моторики желчного пузыря замедляет эвакуацию желчи, что на фоне резкой смены рациона и потери веса способствует камнеобразованию. По некоторым данным, пороговым значением для поддержания эффективного опорожнения желчного пузыря является потребление жира не более 7–10 г/сут. В исследовании Йохансон K. (Johansson K.) и соавт., в которое вошли более 8 тысяч пациентов, участники были поделены на две группы: первая придерживалась VLCKD (500 ккал в сутки), вторая — LCKD с ограничением потребления углеводов и суточной калорийностью 1200–1500 ккал [90]. Потеря веса за год была больше в группе VLCKD (-11,1 против -8,1 кг; 95% ДИ -3,1: -2,4; р<0,001). Авторы пришли к выводу, что риск развития калькулезного холецистита, требующего госпитализации или холецистэктомии, на фоне КД низкий, однако у пациентов, придерживавшихся VLCKD, он был в 3 раза выше, чем при потреблении 1200–1500 ккал [90].

Микробиота

В метаанализе данных 6 когортных исследований и 3 in vivo было продемонстрировано, что КД влияет на микробиом кишечника [91]. Опубликованные наблюдения этих изменений носят противоположный характер. Также авторы подчеркивают, что состав микробиоты может сильно варьироваться, а ее пластичность может зависеть от большого количества факторов, в том числе питания в прошлом. Необходим поиск наиболее эффективных моделей КД, в том числе с добавлением пре- и пробиотиков, количество которых, как было отмечено, резко снижается на фоне КД [91] (табл. 8).

**Table:** Таблица 8. Исследование влияния КД на работу желудочно-кишечного тракта

Автор	Длительность, мес	Средний возраст, лет	Количество участников, n	Побочные эффекты	Оценка качества жизни	Оценка состава тела	Снижение веса	Вывод авторов
Paoli А. и др., 2019 г. [91]	0,2–6	1–50	n=120. 6 РКИ	Явления диспепсии	Не описано	Не описано	Не описано	Полученные результаты изучения микробиоты противоречивы и требуют дальнейшего изучения
Johansson K. и др., 2013 г. [90]	12	46	n=8361. ИМТ 33,4 кг/м2 Ожирение: I ст. — 51%, II ст. — 23%, III ст — 8%. Случайно разделены на 2 группы: 1) VLCD и суточной калорийностью 500 ккал (n=3773), 2) LCKD и 1200–1500 ккал (n=4588). ПССПв 1-й группе у 1,8%,во 2-й — у 3,3%	В течение 6361 человеко-лет в 1-й группе выявлено n=48 камней в желчном пузыре, во 2-й группе —14 (152 против 44/10 000 человеко-лет;коэффициент опасности 3,4, 95% ДИ 1,8–6,3; P<0,001). Из 62 событий, связанных с наличием камней в желчном пузыре, 38 (61%) привели к холецистэктомии (n=29 в 1-й группе,n=9 — во 2-й;отношение рисков 3,2, 95% ДИ 1,5–6,8; P=0,003)	Не описано. Исследование завершили 82% в 1-й группе и 78% во 2-й	Не описано	Потеря веса за год была больше в группе VLCKD (-11,1 против -8,1 кг; 95% ДИ -3,1–-2,4; р<0,001)	Риск развития калькулезного холецистита, требующего госпитализации или холецистэктомии, на фоне КД низкий, однако у пациентов, придерживавшихся VLCKD, он был в 3 раза выше, чем при потреблении 1200–1500 ккал

У пациентов с сопутствующей патологией органов ЖКТ необходимо рассмотреть возможность дополнительного введения пищевых волокон и инструментальных методов обследования еще до старта КД.

Мочевыделительная система

Дегидратация

Основными симптомами обезвоживания на фоне КД являются сухость во рту, головные боли, эпизоды головокружения, снижение остроты зрения и ортостатическая гипотензия. Помимо дегидратации, причиной подобных жалоб также могут быть гипонатриемия и гипомагниемия в результате усиления экскреции КТ с мочой. В связи с чем при соблюдении КД, особенно на первых этапах, рекомендовано потребление не менее 2 л жидкости в день. При выраженной гипотонии возможно увеличение потребления поваренной соли и соблюдение питьевого режима (не менее 2 л в сутки). Головная боль, как правило, длится первые несколько дней после перехода на КД. Следует заметить, что КД весьма успешно применяется для лечения хронической мигрени, однако пациенты должны быть предупреждены о возникновении подобных негативных реакций [92].

Мочекаменная болезнь

В ряде исследований были получены данные о высоком риске развития мочекаменной болезни (МКБ) на фоне соблюдения КД [18]. Примечательно, что наиболее часто МКБ регистрировалась в молодом возрасте, а также при наличии отягощенного семейного анамнеза и повышении соотношения Ca/Cr (креатинин) в моче >0,2. Анализ показал, что в составе камней преобладают мочевая кислота, оксалаты кальция или смеси оксалата кальция и фосфата кальция/мочевой кислоты. Развитие подобного осложнения связывают с хроническим ацидозом, дегидратацией и усилением мальабсорбции жиров. При наличии отягощенного анамнеза по МКБ рекомендуется периодический контроль общего анализа мочи перед началом протокола.

Почечная недостаточность

Безопасность применения КД у пациентов с сопутствующими заболеваниями почек часто вызывает ряд вопросов относительно рисков развития почечной недостаточности вследствие возможного увеличения потребления белковых продуктов. Итальянские ученые провели проспективное исследование с участием 92 пациентов, использующих VLCKD в течение 3 мес [92]. Тридцать восемь пациентов на момент включения в исследование имели ХБП 1–2 стадии, а остальные 54 условно были отнесены к группе контроля, так как скорость клубочковой фильтрации была в норме. Авторы сообщают об отсутствии какой-либо отрицательной динамики почечной функции. Интересен тот факт, что результаты обследования 27,7% пациентов с почечной недостаточностью значимо улучшились через 3 мес соблюдения КД. Однако в связи с тем, что в настоящее время данных о результатах влияния более длительного применения КД на мочевыделительную систему нет, назначение низкоуглеводного рациона пациентам с сопутствующей патологией почек должно сопровождаться тщательным контролем со стороны специалистов (табл. 9).

**Table:** Таблица 9. Исследование влияния КД на работу мочевыделительной системы

Автор	Длительность, мес	Средний возраст, лет	Количество участников, n	Побочные эффекты	Оценка качества жизни	Лабораторные данные	Оценка состава тела	Снижение веса	Вывод авторов
Bruci A. и др., 2020 г. [93]	3,7±2	51,3±12,2 1) 47,96±12,97 2) 55,97± 9,3	n=92. ИМТ 33,8±5,8 кг/м2. Разделены на 2 группы: 1) СКФ от 60 до 89 мл/мин/1,73 м2, 2) СКФ ≥90 мл/мин/1,73 м2	В первые дни: запор, диарея, спазмы в животе, тошнота, усталость, голод и головокружение. 7,7% пациентов — улучшение функции почек в конце диетического вмешательства	Не описана	СКФ до КД 94,46±18,75мл/мин/1,73 м2,после — 95,75±18,52 мл/мин/1,73 м2 HbA1с до КД 5,65±0,81%,после —5,33±0,39%. ЛПНП до КД 120,17±42,92 мг/дл, после —117,38±38,65 мг/дл. ТГ до КД 156,44±90,87 мг/дл, после —102,62±35,71 мг/дл. Белок в моче до КД 12,43±9,50 мг/дл,после — 11,03±7,15 мг/дл	До КД жировая масса — 35,63±9,93, после 24,40±9,00	1-я группа — на 16,27±3 кг, 2-я группа — на 14,64±3,9 кг	КД является безопасным и эффективным диетическим вмешательством у пациентов с ожирением, страдающих легкой формой ХБП, требует медицинского наблюдения. При скрининге следует обратить внимание на уровень микроэлементов и изменение метаболизма костной ткани, а также контролировать объем потребления белка

Использование КД при наличии патологии почек требует периодического контроля, в том числе общего анализа мочи и определения доли белков в рационе.

## ЗЛОКАЧЕСТВЕННЫЕ НОВООБРАЗОВАНИЯ

Учитывая закономерное снижение синтеза инсулина и ИФР-1 на фоне КД, в настоящее время активно изучается ее возможное применение у пациентов с онкопатологией [93]. Большинство авторов исключают прямое влияние КД на канцерогенез. Предполагается, что именно сочетание КД со стандартными схемами лечения может позволить снизить дозы препаратов и даже проявления побочных эффектов. Шей M. (Hsieh M.) и соавт. продемонстрировали замедление роста плоскоклеточной карциномы на КД in vivo в сочетании с цитотоксической терапией, вероятно, за счет снижения экспрессии рецепторов GLUT1 [94]. КД оказывает выраженное противоопухолевое действие в модели ксенотрансплантата на мышах, возможно, регулируя аутофагию клеток. Представляет интерес изучение влияния КД при раке молочной железы на фоне терапии рапамицином, одним из побочных эффектов которого считается развитие гипергликемии. Контроль состояния углеводного обмена на фоне КД привел к снижению роста и дифференцировки раковых клеток у мышей, вызванных глюкозой.

Опубликованные данные доклинических и клинических исследований подтверждают определенный потенциал улучшения качества жизни таких пациентов при соблюдении КД, что позволяет рассмотреть данный рацион в качестве дополнительной меры. Однако отсутствие единых протоколов, широкой базы данных наблюдения и оценки отсроченных эффектов требует дальнейшего изучения вопроса.

## ЗАКЛЮЧЕНИЕ

На сегодняшний день не существует единого мнения, позволяющего широко применять низкоуглеводный вариант рациона, несмотря на наличие положительных клинических наблюдений у пациентов с СД 2 типа, ожирением, СПКЯ и различными неврологическими нарушениями. При этом КД становится все популярнее среди лиц даже без сопутствующей патологии, хотя столь радикальное изменение привычного рациона требует строгого медицинского наблюдения не только диетологов, но и врачей других специальностей. Механизм действия, возможные побочные эффекты, в том числе и в отсроченном периоде, возможности применения сопутствующей медикаментозной терапии, особенно при нарушениях углеводного обмена, требуют дальнейшего изучения. Несмотря на то что данные о некоторых преимуществах КД стали публиковаться чаще, определенные опасения по поводу потенциальных рисков и использования в долгосрочной перспективе сохраняются из-за небольшого количества подобных клинических исследований, а также серьезных доводов о возможном негативном влиянии на риски сердечно-сосудистых катастроф, отраженных в выводах последних исследований на большой когорте пациентов. Примечательно, что существует серьезная нехватка руководств по этой теме, а использование и внедрение КД происходят в значительной степени в отсутствие четких доказательных показаний.

## ДОПОЛНИТЕЛЬНАЯ ИНФОРМАЦИЯ

Источники финансирования. Работа была проведена при поддержке государственного задания АААА-А20-120011790162-0.

Конфликт интересов. Авторы декларируют отсутствие явных и потенциальных конфликтов интересов, связанных с публикацией настоящей статьи.

Участие авторов. Все авторы одобрили финальную версию статьи перед публикацией, выразили согласие нести ответственность за все аспекты работы, подразумевающую надлежащее изучение и решение вопросов, связанных с точностью или добросовестностью любой части работы
